# Nitrogen-Controlled Host Gatekeeping: Regulatory Chokepoints Across Four Windows for Diazotroph Access

**DOI:** 10.3390/ijms27073059

**Published:** 2026-03-27

**Authors:** Cassio Carlette Thiengo, Maria Julia Brossi, Carlos Alcides Villalba Algarin, João Vitor Leonel, Lucas William Mendes, Fernando Shintate Galindo, José Lavres

**Affiliations:** 1 Luiz de Queiroz College of Agriculture (ESALQ), University of São Paulo (USP), Piracicaba 13418-900, SP, Brazil; carlosvillalba@usp.br; 2 Department of Plant Science, College of Agricultural Sciences, The Pennsylvania State University, University Park, State College, PA 16802, USA; majubrossi@psu.edu; 3 Centro de Investigación Capitán Miranda, Instituto Paraguayo de Tecnología Agraria, Route VI, Km 21.5, Capitán Miranda, Itapúa 070504, Paraguay; 4 Center for Nuclear Energy in Agriculture, University of São Paulo, Piracicaba 13416-000, SP, Brazil; jvitorleo@usp.br (J.V.L.); lwmendes@cena.usp.br (L.W.M.); jlavres@usp.br (J.L.); 5 Faculty of Agricultural and Technological Sciences, São Paulo State University (UNESP), Dracena 17900-000, SP, Brazil; fernando.galindo@unesp.br

**Keywords:** nitrate sensing, rhizosphere microdomains, host carbon energy arbitration, biological nitrogen fixation, host permissiveness

## Abstract

Although diazotrophy is compatible with low-carbon agriculture and aligned with sustainability goals, its benefits could be expanded by better leveraging associative plant–diazotroph partnerships. Host control, however, remains underemphasized despite increasing resolution of microbial determinants of colonization. Understanding how plants tune permissiveness across fluctuating mineral N landscapes is therefore central to explaining when microbial presence translates into measurable diazotrophic function and plant N gain. Here, we propose an N-mediated host gatekeeping framework that organizes existing evidence into four licensing windows: (i) spatial positioning of permissive sites, (ii) N-sensitive transcriptional thresholding, (iii) local immune tuning at the contact interface, and (iv) carbon energy arbitration sustaining fixation and N transfer. Our model predicts that moderate, spatially heterogeneous mineral N biases the root interface toward permissive states in which microdomain colonization can translate into measurable biological nitrogen fixation, whereas at either extreme one or more windows tend to close. In crops, soil heterogeneity and genotype-linked root functional traits act as filters shaping when functional engagement becomes possible. By reframing N as both a resource and a signal acting through host arbitration, this model clarifies how permissiveness can be tuned to better realize diazotrophic potential and support plant N gain under rational mineral N management.

## 1. Introduction

Biological nitrogen fixation (BNF) represents a major natural N input to terrestrial ecosystems and occurs at agronomically relevant scale in cultivated lands [1], yet it remains underused as a predictable route to reduce reliance on synthetic N fertilizers. This limitation is consequential as modern agriculture still sustains high yields through mineral N inputs despite low retention efficiency in many systems, with losses frequently exceeding half of applied N and causing well-documented impacts on water quality, air pollution, and climate forcing [2,3,4]. Consequently, dominant mitigation strategies have centered on improving fertilizer-use efficiency through management, while root-associated biological sources remain peripheral. This persists in part because mineral N itself strongly governs diazotrophic activity and constrains the predictability of plant benefit [5,6,7].

BNF is inherently energy-intensive, requiring substantial reductant and ATP to sustain nitrogenase activity [8]. In cropping systems, plant-associated N fixation occurs through three broad routes: (i) highly specialized legume–rhizobium symbioses with nodule organogenesis [9], (ii) opportunistic endophytic associations in non-legumes that establish intra- and intercellular niches and can transfer reduced N under specific conditions [10], and (iii) rhizosphere or rhizoplane consortia in which chemotaxis, biofilms, and root uptake processes shape diffusive N delivery to the host [11]. Whereas endosymbiosis fixation can, in some cases, meet most of the host’s N requirement, associative interactions can still provide a meaningful share of plant N in crops, with reported contributions on the order of ~5–30%. Furthermore, across systems, diazotrophic activity is most evident under low or limiting N supply and typically declines as mineral N inputs increase [12,13,14].

Most mechanistic explanations for this variability have focused on the microbial side of plant–diazotroph interactions. Mineral N gradients reprogram early microbial behaviors, including chemotaxis, motility and the active targeting of exudate-rich domains at the rhizoplane [15,16]. Once cells reach the root interface, these shifts can carry forward into attachment and anchoring processes that stabilize contact [17,18], as well as into persistence and morphological adjustments that keep populations poised until N conditions become permissive again [19]. At the functional level, mineral N directly tunes nitrogenase gene expression and activity through conserved regulatory modules (e.g., NifA–NifL system and PII-family signaling) [20,21]. Beyond cell-level regulation, chronic N inputs can reduce soil microbial biomass and dampen temporal community turnover, often affecting diazotroph taxa [22]. Although this body of work provides detailed insight into microbial regulation, it does not, on its own, explain when colonization culminates in functional associations that deliver a measurable N return to the host.

As a second, equally important, layer, host arbitration of diazotroph association remains poorly resolved. The root microbiome is often framed as a “second genome” [23], emphasizing integration but often leaving host decision-making implicit. The cry-for-help concept offers one entry point, proposing that shifts in root exudation recruit beneficial microbes, including diazotrophs, as N becomes limiting [24,25]. However, accumulating evidence supports the idea that mineral N regulates host permissiveness through rapid, local decisions rather than a single global switch. Changes in nitrate status can quickly reconfigure signaling and hormone crosstalk, including nitrate-regulated auxin dynamics, and thereby bias where and when roots become transiently receptive to close association [26,27]. When recruitment occurs, functional engagement likely depends on whether carbon and energy economics can sustain support under fluctuating N supply and on whether immunity is locally tuned under buffered redox control at the interface [28,29]. Across species and environmental contexts, microbial colonization is actively filtered by the host [20,30], yet plant control is still often treated as permissive by default rather than as conditional, spatially and temporally bounded decisions that must align for measurable N return.

To render this host decision logic tractable, we formalize an N-controlled host-licensing framework that organizes fragmented evidence into four sequential windows that gate host permissiveness once microbes reach the soil–root interface. These windows represent conditional chokepoints that integrate local host decisions, N signaling, and energetic cost from encounter through post-contact: (I) rapid tuning of epidermal sensitivity through NO_3_^−^ sensing, auxin redistribution, and Ca^2+^ signaling via NRT1.1/CHL1; (II) positioning of N-sensitive transcriptional thresholds, in legumes exemplified by NLP-NIN crosstalk; (III) stabilization of entry sites via CBL-CIPK control with local redox regulation and NO/ROS dynamics; and (IV) carbon and energy arbitration through SnRK1-TOR balance, which determines when sustaining BNF pays off relative to mineral N assimilation.

## 2. Host-Arbitrated Licensing Windows Under Nitrogen Control

We define licensing as the plant physiological state that permits, tolerates, and sustains diazotroph partnership, achieved when four partially independent windows align in the same space and time.

### 2.1. Window I—Positioning Permissive Sites: NRT1.1, Ca^2+^ Pulses, and Auxin

Nitrate signaling is the best-characterized N pathway in plants and is triggered within seconds to minutes after contact with NO_3_^−^ at the soil–root interface [31]. Several recent syntheses place NRT1.1/NPF6.3 at the core of a nitrate signaling network that links uptake, primary response, and root architectural plasticity, highlighting its dual role as both transporter and sensor [32]. Mechanistically, NO_3_^−^ entry elicits Ca^2+^ signatures that are decoded by CPK10/30/32 and coupled to NRT1.1/CHL1 (NPF6.3) within the Ca^2+^–CPK–NLP backbone, in which phosphorylated NLPs rapidly reprogram genes associated with transport, assimilation, and root growth [33,34]. NPF6 orthologues have been described across multiple crops, and in *Arabidopsis*, NRT1.1/NPF6.3 provides the prototype in which adjustments in transporter activity and localization reprogram auxin flow in lateral root primordia [35,36]. This integration tunes PIN positioning and the hormonal gradient along the root axis, such that NO_3_^−^ oscillations are translated into auxin redistribution. The effective nitrate ranges and gradients vary across species, cultivars, and nutritional status, recalibrating the sensitivity of this system and the threshold at which the root responds with architectural plasticity [26,37].

Under low and diffuse NO_3_^−^, NRT1.1 favors auxin efflux out of primordia, restricting lateral root emergence and elongation; the tissue remains in a conservative state, with few exploration foci and a limited number of permissive sites along the axis [38]. Under high and homogeneous NO_3_^−^, the local Ca^2+^–auxin axis integrates sufficiency cues and reduces lateral root density and extension, because foraging demand is low and growth concentrates into a more compact architecture [39]. At the whole-plant scale, these local adjustments echo the concept of preferential nitrate foraging, whereby lateral roots proliferate in NO_3_^−^-rich patches in response to systemic demand signals, integrating local supply with global N status [40,41]. By contrast, when NO_3_^−^ occurs at moderate levels and in a heterogeneous distribution, release of the brake on auxin transport allows primordia to advance in NO_3_^−^-rich sectors, consolidating cell wall/apoplast microdomains that become anatomical permissiveness points [42,43]. These anatomical points form the spatial map on which, in Windows II–IV, the root can couple the transcriptional, immune, and energetic thresholds that define the effective licensing of the diazotrophic partnership.

### 2.2. Window II—Nitrogen-Sensitive Transcriptional Thresholds (NLPs and NIN in Legumes)

In Window II, at the permissive sites anatomically established in Window I, the root decides locally, within the nuclei of epidermal and cortical cells exposed to apoplastic NO_3_^−^, whether that focus enters the mineral assimilation route or the nodulation program. NLPs (NIN-Like Proteins) read NO_3_^−^ and activate, within the same nuclear compartment, promoters controlling uptake, assimilation, and metabolic adjustment [44]. Recent syntheses consolidate NLPs as transcriptional hubs of the nitrate response and central integrators of NO_3_^−^ signals, positioning this module as a logical interface between N availability and the transcriptional reprogramming of root growth [45,46]. In parallel, NIN (Nodule Inception) responds to LCOs (Nod factors) perceived in the epidermis and coordinates infection and nodule formation in the adjacent cortex [47]. Recent reviews place NIN at the center of the nodulation circuit, integrating LCO perception, nodule organogenesis, and autoregulation pathways [48]. In *Lotus japonicus*, mechanistic evidence confirmed that the Nod factor receptors NFR5 and NFR1 form the LCO/Nod receptor complex, and that a juxtamembrane motif in NFR5 is essential for signaling and assembly [49]. As the apoplastic NO_3_^−^ pattern shifts, it repositions the NLP–NIN transcriptional threshold within these nuclear foci and defines three decision states.

When NO_3_^−^ is low and diffuse, the apoplast provides a weak signal, NLPs accumulate only minimally in the nuclear compartment, and local permissiveness remains low, meaning that few microdomains are effectively responsive. Symbiotic competence persists, but NIN retains a short local reach even when LCOs are present [50]. Although the classical literature emphasizes inhibition of nodulation under high NO_3_^−^, work on N economy and the energetic costs of symbiosis suggests that extremely low N states can also restrict nodulation through limited growth and limited reserves required to sustain functional nodules, indicating that an optimal symbiotic window lies between these extremes [51,52].

Under moderate and heterogeneous NO_3_^−^, with microgradients or pulses at the rhizoplane, these same nuclear foci co-activate NLPs without suppressing early infection and nodulation components [53]. If LCOs impinge on the corresponding epidermal microarea, NIN is induced within the same tissue, sensitivity to LCOs is maintained, and adhesion and entry advance within these microdomains, which become transcriptionally licensed sites for symbiosis [33,54]. Studies in *Lotus japonicus* and *Medicago truncatula* indicate that the physical and functional interaction between NLPs and NIN, together with their integration with CLE peptides and the autoregulation of nodulation (AON) pathway, is central to this permissive regime under moderate N [55,56].

When NO_3_^−^ is high and homogeneous, the apoplast sustains a strong signal, NLPs continuously occupy the nuclear compartment, the threshold for LCO/Nod responsiveness rises within the same transcriptional focus, early symbiosis genes retract, and the decision shifts towards systemic mineral assimilation [56,57]. In this scenario, NO_3_^−^ inhibition of nodulation emerges from multiple layers, spanning NLP–NIN modules and extending to shifts in flavonoids, defense pathways, and redox status in infected roots [55]. In *Parasponia andersonii*, a non-legume that controls symbiosis, nodulation was suppressed under high N and CLE genes (PanCLE2/8/9) were induced in roots and nodules, reinforcing the integrated action of NO_3_^−^ and AON in restricting nodules under N sufficiency [58].

Thus, at the permissive sites anatomically defined in Window I, Window II positions, via the NLP–NIN axis and its modulators, the transcriptional threshold that determines whether a given focus enters the nodulation circuit or remains committed to the NO_3_^−^ assimilation route.

### 2.3. Window III—Permissiveness Without Immunosuppression

Plant innate immunity is often described as two coupled modules. The first corresponds to pattern-triggered immunity, PTI, in which cell-surface pattern-recognition receptors detect conserved microbial motifs and assemble a basal surveillance response [59]. The second corresponds to effector-triggered immunity, ETI, in which intracellular NLR-type receptors detect effectors and amplify this response into a second layer of detection and control [60]. Although this framework was delineated primarily in plant–pathogen systems [61], recent work emphasized that the same modules also structure root interaction with beneficial microorganisms in the rhizosphere and rhizoplane, with distinct outcomes that include compatible colonization, induced resistance, and microbiome remodeling [62,63].

At foci that pass Windows I and II, the licensing decision shifts to this surface-immunity layer. Here, PRR–PTI architecture described above operates in the epidermis and outer cortex, modulating how the root reads microbial signals at the rhizoplane and in the apoplast [64]. PRR activation triggers the canonical PTI cascade, including rapid ionic reprogramming, Ca^2+^ and MAPK signaling, reactive oxygen species production, and focal cell-wall reinforcement [65,66]. When effectors are perceived, NLRs activate ETI, which amplifies and prolongs the response [60,67]. In Window III, the host seeks to maintain this module in a pulsatile and spatially restricted PTI regime, sufficient for surveillance and local containment, yet still compatible with adhesion and controlled entry at the contact site [68,69].

Fine-scale regulators determine whether a permissive microdomain remains accessible or becomes durably blocked. The CBL–CIPK complex acts as one such regulator and operates on the same Ca^2+^ signatures and membrane potential to tune the amplitude and duration of the response at the contact site [70,71]. Nitric oxide (NO) further illustrates the bifurcation between defense and remodeling: in the *Azospirillum brasilense*–tomato interaction, NO promotes lateral root development from initially defensive microdomains [72]. More recent studies also show that apoplastic redox sinks, expressed in Asc/DHA and GSH/GSSG ratios and in peroxidase activity, favor this recoding by shortening the duration of oxidative signals and keeping the response in brief pulses [73,74].

Within the proposed conceptual model, N acts at this level as a background chemical modulator. The form and microdistribution of N in the apoplast tune pH and peroxidase activity and, therefore, the persistence of redox signals, consistent with models that treat apoplastic pH as an integrator of N nutrition, redox state, and root signaling [75,76]. Moderate and spatially restricted NO_3_^−^ tends to buffer pH and limit peroxidase activity, favoring short and reversible oxidative signals, whereas accumulated NH_4_^+^ acidifies the apoplast, stabilizes wall reinforcement, and is associated with more prolonged and costly responses [77,78]. When defense enters this prolonged state, wall reinforcement becomes a structural barrier, extracellular oxidative signaling remains elevated, and membrane trafficking is reprogrammed, with PRR endocytosis and redistribution of surface components [79,80]. Under this condition, the microdomain functions predominantly as a barrier site and tends to cease being a candidate to sustain carbon flux and BNF activity in Window IV.

Partner-emitted signals refine this tuning without disabling surveillance. In legumes, LCOs attenuate PTI and synchronize the local redox pulse with the transcriptional license achieved in Window II [81]. In non-legumes, exopolysaccharides perceived by EPR3, quorum-sensing AHLs, and small phenolics attenuate PTI in a strictly local manner and improve adhesion, converging on a state of vigilant tolerance at the permissive site [82,83]. These microdomains, simultaneously licensed by Windows I and II and maintained in an immune-permissive regime in Window III, constitute the candidates that can advance to the energetic arbitration of Window IV.

### 2.4. Window IV—Energetic Arbitration

Once a microdomain is permissive from anatomical, transcriptional, and immune standpoints, as established in Windows I–III, the decision is no longer whether the partner enters, but how much C the host is willing to deliver to the microbial consortium. In Window IV, host energetic arbitration determines whether BNF becomes functional or whether the focus returns to operating as a site of mineral assimilation [84].

The SnRK1–TOR axis functions as a central node of this arbitration. SnRK1 responds to deficit states, signaled by low sucrose and ATP status and by trehalose-6-phosphate-related cues, and activates programs of conservation and reserve mobilization [85]. By contrast, TOR, together with its effector S6K, responds to metabolic abundance and promotes host anabolism [86]. In model species, this module is described as a regulator of C allocation and source–sink relations, modulating translation and the synthesis and degradation of reserves according to energetic status [87,88]. In addition, studies in roots and growing tissues link variation in N supply and NO_3_^−^/NH_4_^+^ assimilation to shifts in the SnRK1-TOR balance, reinforcing the coupling among N status, C metabolism, and growth decisions [57,89]. At the root–microorganism interface, this balance determines how much C allocated to host growth and how much can be redirected to the fixing consortium within a permissive focus.

Energetic arbitration is expressed at the cell–apoplast interface through the exposure, activity, and retrieval of sucrose transport capacity. H^+^ gradients generated by plasma-membrane H^+^-ATPases shape the electrochemical environment in which these transporters operate, while vesicular trafficking determines which transporters remain present at the permissive focus [90]. SWEET family sucrose transporters facilitate efflux into the apoplast [91], whereas SUT/SUC high-affinity H^+^-sucrose symporters recapture and redirect C toward the microbial interface, here conceptualized as an adherent biofilm comprising fixing cells and supportive commensals [92,93]. When the SnRK1-TOR balance permits sustained carbon donation, cycles of transporter exposure and recycling maintain localized sucrose sinks characteristic of nodules and other symbiotic interfaces [94].

At the wall and intercellular matrix scale, sucrose flux is converted into microaerobic respiratory support for the consortium. This framing is consistent with evidence that functional nodulation depends on continuous C supply to sustain intense respiration under low O_2_ availability [95]. Studies of root growth and signaling further show that coordinated oscillations of pH, reactive oxygen species, electrochemical gradients, and C supply typify active interfaces of exchange and remodeling [90,96]. When these thresholds cease to be met, the interface stops operating as a zone of active exchange and converges towards the barrier state described in Window III, with a sharp reduction or interruption in C delivery to the consortium [97,98].

The NO_3_^−^ and NH_4_^+^ patterns established in Window III reappear here as modulators of the energetic balance at the permissive focus. Moderate and spatially restricted NO_3_^−^ favors an SnRK1–TOR equilibrium compatible with sucrose provision to the consortium by sustaining buffered apoplastic pH and an N demand that justifies the C cost. By contrast, high and homogeneous NO_3_^−^, or elevated NH_4_^+^, is associated with N sufficiency states that shift the axis towards TOR-dominated regimes, reinforce mineral assimilation, and reduce the incentive to maintain C flux towards BNF [57,89]. Under these conditions, the marginal gain of maintaining BNF tends not to compensate for the C cost, and the permissive focus progresses towards a self-growth-dominated state, with lower probability of sustaining an energetically favorable interface for fixation.

Under energetically and spatially favorable regimes, partner-emitted signals refine this tuning without switching off peripheral surveillance. Exopolysaccharides, LCOs, and small metabolites synchronize the exposure and recycling of SWEET and SUT or SUC at the contact site, reinforce microaerobiosis within the biofilm, and preserve a ring of active defense around the functional focus [99,100]. In summary, when energy status, and N form (and some other factors, such as oxygen constraints and cofactor availability) converge within a microdomain that has surpassed the prior thresholds, the host sustains a C flux compatible with BNF and the focus stabilizes as a functional partnership site. If this convergence fails, energetic arbitration favors mineral assimilation, C delivery retracts, and the permissive window closes at that site.

**Figure 1:**
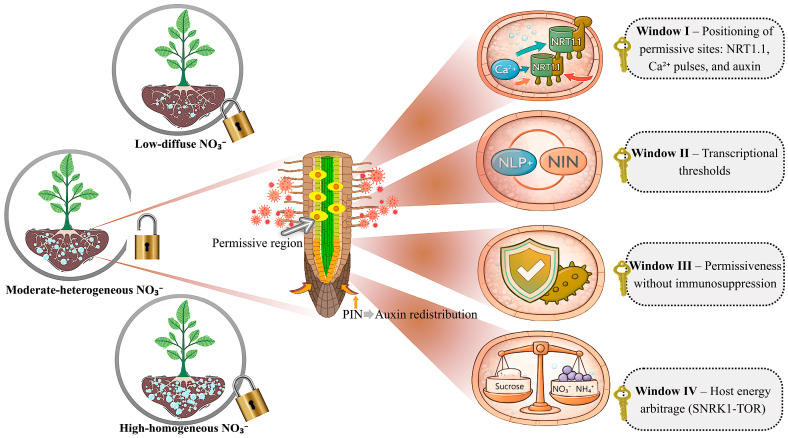
Conceptual model of sequential host license windows that position permissive sites (NRT1.1/CHL1), establish nitrogen-sensitive transcriptional thresholds (NLP–NIN), maintain contact-site permissiveness without immunosuppression, and arbitrate carbon allocation for BNF through SnRK1-TOR axis. The longitudinal root schematic highlights how diazotroph encounter coincides with local PIN–auxin patterning to define permissive microdomains that initiate Window I and provide the spatial substrate for Windows II–IV. Extreme N regimes (very low and diffuse N, or high and homogeneous mineral N) constrain progression, whereas moderate and spatially heterogeneous NO_3_^−^ favors window convergence and functional diazotrophy.

For clarity, Figure 1 focuses on the moderate-N regime, which represents the unique configuration in which all four licensing windows remain simultaneously open and diazotrophy becomes functionally viable.

## 3. From Model Plants to Crops: Consistent Patterns Across Scales and Limits to Generalization

We next ask whether the four-window framework leaves a consistent signature in crops across experimental scales (Supplementary Table S1 provides additional supporting examples alongside those discussed below). A substantial body of evidence is coherent with a shared logic in which host genotype sets physiological ceilings, while mineral N modulates the local thresholds that determine when and where colonization becomes functionally measurable BNF. In cereals, particularly in rice, ammonium nitrate fertilization shifted root-associated diazotroph communities, with responses tracking genotype [101]. In sorghum grown on Cerrado soil, contrasting N rates (12 vs. 120 kg N ha^−1^) reorganized nifH diversity and identity in the rhizosphere, with N rate emerging as a primary determinant while cultivar dependence persisted [102]. Field observations in maize similarly indicated that N addition reduced diazotroph populations during early growth even though roots remained the preferred colonization niche, implying raised establishment thresholds rather than loss of habitat suitability [103]. Sugarcane extended this pattern to endophytism, with reduced colonization of *Gluconacetobacter diazotrophicus* under a high-N regime (~6.3 mM vs. 44.8 mM) [104]. Together, these responses are broadly consistent with Windows I and II, in which external N sensing and host regulatory filtering shift the permissive threshold for colonization and early diazotroph engagement.

When the question shifts from “*did it colonize*?” to “*did it fix N actively*?”, host ceilings become more explicit and the mineral N regime more decisive. In switchgrass maintained for five years under 0, 90, and 180 kg N ha^−1^ yr^−1^, nitrogenase activity was highest in roots under zero N and minimal or undetectable at the highest N level, accompanied by reconfiguration of expressed *nifH* OTUs, consistent with functional suppression under N sufficiency even when part of the microbiota remains detectable [105]. In line with this, elevated mineral N suppressed measurable fixation even when C supply via exudates was maintained, indicating that mineral N can override carbon inputs that would otherwise sustain diazotroph activity around roots [106].

Some host genotypes and structural traits appear able to sustain local interface conditions compatible with diazotroph function outside “ideal” macro-environmental regimes. In the Sierra Mixe maize landrace, a carbohydrate-rich, microaerobic niche provided by mucilage on aerial roots supported nitrogenase activity and ^15^N_2_ incorporation, linking functionality to local C provision and O_2_ restriction [107]. Classical wheat studies linked genotypic variation in exudation to higher associative fixation and greater transfer of fixed N to the host (13–17% of root N and 2.9–3.9% of shoot N) [108]. In native South American C4 grasses, free-living and associative diazotroph contributions were estimated at 22% to 36% of total plant N using ^15^N natural abundance. Colonization increased over time but was suppressed by N fertilization, and fixation potential varied among species with distinct growth strategies, with tussock-forming grasses showing higher contributions [109].

Comparability across experiments improves substantially when molecular and sequencing evidence is paired with ^15^N isotopic tracing approaches. Sequencing resolves community identity and functional engagement, while isotopes quantify how much fixed N reaches the host [110,111]. In maize under co-inoculation, BNF gains, and N-use efficiency were maintained at intermediate mineral N but declined at the highest regime across a 0, 120, and 240 kg N ha^−1^ gradient [112]. In *Urochloa brizantha* cv. Marandu inoculated with *Herbaspirillum seropedicae*, the fixed-N contribution declined from 21.5% under zero N to 8.6% under high N, consistent with a shift in the host cost–benefit balance away from fixation [113]. Soybean remains the canonical agronomic success case in Brazil. Elite *Bradyrhizobium* spp. inoculation often obviates mineral N, yet the system still draws on soil pools and or organic matter mineralization [114,115], and even 20 kg N ha^−1^ reduced the fraction derived from the atmosphere by >20 percentage points [116]. Mechanistically, this sensitivity reflects host arbitration that integrates local infection and nodule development with whole-plant resource allocation and long-distance signaling [117,118]. These functional responses are consistent with Windows III and IV, where carbon economy, host reward allocation, and whole-plant nutritional coordination determine whether colonization is translated into measurable N fixation and host benefit.

The limits of generalization become most apparent when experimental scales and durations are compared. In model and in vitro systems, “deprivation”, “moderate”, and “high” N are operational categories with well-defined boundaries, whereas in soils plants rarely experience true N absence because organo-mineral reservoirs sustain background supply and edaphic controls continuously reshape availability [119]. Apparent inoculation gains under high N doses likely reflect rhizosphere microenvironments generated by soil heterogeneity, yet these local states are seldom measured directly, leaving part of the response effectively stochastic at current experimental resolution. Over longer timescales, chronic N fertilization reduces soil N_2_-fixation potential and drives persistent reconfiguration of diazotrophic communities, rather than merely transient pulse responses [120,121].

**Figure 2:**
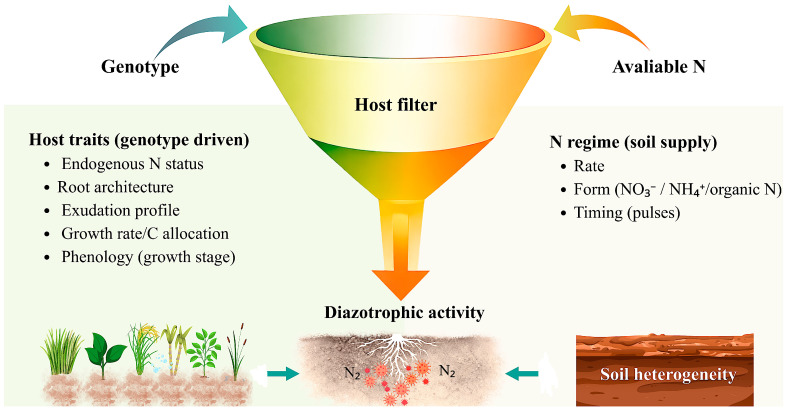
Conceptual funnel summarizing how crop-scale diazotrophic activity emerges from the interaction between a genotype-defined host filter and the prevailing N regime (soil supply). On the host side, genotype-driven traits, including root architecture, endogenous N status, exudation profile, growth rate/C allocation, and phenology, shape permissiveness at the root interface. On the N side, supply rate, chemical form (NO_3_^−^, NH_4_^+^, organic N), and timing (pulses) modulate the local conditions experienced by roots, with soil heterogeneity further partitioning these regimes into microsites. Together, these interacting constraints determine whether diazotroph presence is expressed as measurable N_2_-fixing activity across contrasting crop systems.

Taken together, these studies support a coherent framework in crops: responses to diazotrophs are not determined by microbial presence alone, but by the interaction between the mineral N regime at the root–microorganism interface (rate, form, and spatial distribution) and the host filter (genotype and traits), which together condition permissiveness and functional performance (Figure 2).

## 4. Final Remarks and Outlook

The “licensing windows” framework synthesizes and organizes a fragmented body of literature into a sequence of conditional bottlenecks through which N-modulated host arbitration governs when diazotroph–plant associations become functional and can deliver a measurable N return to the host. In our conceptual model, moderate and heterogeneous N is the condition most likely to shift the system towards open windows, because it places the root interface in a permissive state where colonization can translate into function. Across crops and experimental scales, host genotype and other functional traits establish physiological limits for realizing N gains from diazotroph association, while soil mineral N shifts the local thresholds that determine when these limits become restrictive or permissive. Critically, soil heterogeneity generates millimeter-scale mosaics of N availability, and chronic N inputs leave legacies that reshape diazotrophic functional potential. Accordingly, more diagnostic studies increasingly impose structured N regimes (rate, form, timing, placement) and prioritizes function-centered outcomes (source partitioning, isotopic approaches, transfer metrics) that separate colonization from host N gain.

On the microbial side, applied efforts have largely focused on bioinoculant formulation, particularly improving viable cell counts, persistence and shelf life [121]. These advances remain necessary, but they are unlikely to ensure predictable host-level benefits on their own when mineral N environments and host arbitration keep permissive states locally closed at the root interface. Clear opportunities are already emerging to achieve tighter control over diazotroph activation (including decoupling mineral N status from activation and N transfer via orthogonal, engineered control circuits and plant-delivered cues), with rhizopine-like systems as a proof of concept (e.g., [122,123,124]. Here, we suggest that shifting part of the applied focus from diazotrophy in isolation towards host arbitration, and the environmental contexts that keep permissive windows open, will be essential to make diazotroph performance more predictable across association types and N landscapes. By reframing biological nitrogen fixation as a host-arbitrated and spatially contingent process, the licensing windows framework provides a mechanistic basis for interpreting variability in plant–diazotroph outcomes and for designing more realistic strategies to integrate BNF into agricultural systems.

## Data Availability

No new primary datasets were generated.

## Supplementary Materials

The following supporting information can be downloaded at: https://www.mdpi.com/article/10.3390/ijms27073059/s1 [56,125,126,127,128,129,130,131,132,133,134,135,136].
