# Supplementary material for: Nitrogen-Controlled Host Gatekeeping: Regulatory Chokepoints Across Four Windows for Diazotroph Access
Source: Int J Mol Sci. 2026 Mar 27;27(7):3059. doi: 10.3390/ijms27073059 (PMC13073011; doi:10.3390/ijms27073059)
Supplement: Supplementary file 1 [file ijms-27-03059-s001.zip › ijms-4188459-supplementary.pdf]

Table S1. Nitrogen (N) supply and its effects on plant–diazotroph association/colonization across symbiotic contexts. The concentrations are reported as millimolar (mM) of the ionic species supplied (NO_3^- or NH_4^+); when the original study specifies the salt, it is shown in parentheses. If the exact salt/concentration is not explicit in the public text, we indicate "salt —, see Methods" to avoid assumptions.

Association type	N supply (mM; ionic species)	Effect	Affected aspect	Citation
**Legume–rhizobium (*Medicago truncatula* × *Sinorhizobium* sp.)**	10 mM NO_3^- (KNO_3)	Negative (marked decrease in nodule number)	NO_3^- induces MtCLE35 and triggers AON/SUNN, repressing nodulation	[125]
**Legume–rhizobium (*Glycine max* × *Bradyrhizobium* sp.)**	5 mM NO_3^- ($NaNO_3$)	Negative (−56% allocation of ^{14}C to nodules: 9% → 4%)	Rapid and reversible inhibition of carbon supply to nodules under NO_3^-	[126]
**Legume–rhizobium (*Medicago truncatula* × *Sinorhizobium* sp.)**	0–0.5 mM NO_3^- (KNO_3)	Positive/Permissive (↑ initiation/entry)	NLP1 → CLE35 integrates the NO_3^- signal into the AON circuit	[56]
**Associative/endophytic (*Zea mays* × *Herbaspirillum seropedicae*)**	1 mM vs 10 mM NO_3^- (salt —, see Methods)	Positive at 1 mM (↑ ARA) / Negative at 10 mM (↓ ARA)	Moderate NO_3^- elevates nitrogenase activity; high NO_3^- reduces it	[127]

**Legume–rhizobium (*Glycine max* × *Bradyrhizobium* sp.)**	5–10 mM NO_3^- (salt —, see Methods)	Negative (suppressed attachment and infection thread elongation)	Anchoring and infection thread progression	[128]
**Associative/endophytic (*Saccharum officinarum* × *Gluconacetobacter diazotrophicus*)**	25 mM NH_4NO_3; 25 mM NH_4^+ (NH_4Cl); 25 mM NO_3^- (KNO_3)	Negative — reduced colonization; pronounced pleomorphism (NH_4NO_3 > NH_4Cl > KNO_3)	Morphology (pleomorphism) and colonization	[129]
**Associative/endophytic (*Saccharum officinarum* × *Gluconacetobacter diazotrophicus*)**	NH_4^+ (increasing doses, $(NH_4)_2SO_4$) and NO_3^- (increasing doses, $Ca(NO_3)_2$) — mM — (see Methods)	Negative — inhibition of ARA and reduced populations (cultivar × N interaction)	ARA and colonization	[130]
**Mechanism (diverse diazotrophs; no plant)**	Elevated NH_4^+ (excess intracellular N)	Transcriptional repression via NifA–PII ⇒ ↓ nitrogenase activity	nif regulation (NifA–PII)	[131]
**Associative/endophytic (*Herbaspirillum seropedicae; in vitro*)**	~18–20 mM NH_4^+ (≈1.0 $g \cdot L^{-1}$ NH_4Cl)	Marked decline in ARA; NH_4^+ "switch-off" (NifA/PII)	Nitrogenase activity (ARA)	[132]
**Associative/endophytic (*Herbaspirillum seropedicae*)**	NH_4^+ (0 → high gradient))	Dose-dependent depression of ARA (NifA/PII discussed)	Nitrogenase activity (ARA)	[133]

**Associative (*Azospirillum brasilense*; in vitro)**	NH_4^+ (pulse, minutes) — mM — (see Methods)	Post-translational “switch-off” (Fe-protein ADP-ribosylation; DraT/DraG) — reversible	Nitrogenase activity (post-translational)	[134]
**Associative/endophytic (*Zea mays* × *Herbaspirillum seropedicae*)**	0.3 mM vs 3.0 mM NO_3^-	Low N (0.3 mM) favors association (↑ nif); 3 mM alters hormonal/morphological responses	23S qRT-PCR (colonization); dual RNA-seq; nifA/nifH/nifW	[135]
**Legume–rhizobium (*Glycine max* × community; split-root)**	~14.3 mM NO_3^- (KNO_3)	Negative — reduced nodulation and BNF; ↓ sucrose/starch; PBM damage; mitigated by enhanced C supply	Carbon supply; symbiosome/PBM integrity; nitrogenase activity	[136]

Abbreviations: AON, autoregulation of nodulation; ARA, acetylene reduction assay; PBM, peribacteroid membrane; ROS, reactive oxygen species. Classification of N levels used elsewhere in the manuscript—Low: 0–1 mM; Moderate: ~1–2.5 mM; High: ≥5–10 mM—is provided for orientation but was not enforced here when authors used multiple regimes in a single experiment.
